# Triiodothyronine (T_3_) upregulates the expression of proto-oncogene *TGFA* independent of MAPK/ERK pathway activation in the human breast adenocarcinoma cell line, MCF7

**DOI:** 10.20945/2359-3997000000114

**Published:** 2019-03-18

**Authors:** Tabata M. Silva, Fernanda C. F. Moretto, Maria T. De Sibio, Bianca M. Gonçalves, Miriane Oliveira, Regiane M. C. Olimpio, Diego A. M. Oliveira, Sarah M. B. Costa, Igor C. Deprá, Vickeline Namba, Maria T. Nunes, Célia R. Nogueira

**Affiliations:** 1 Universidade Estadual Paulista Universidade Estadual Paulista Faculdade de Medicina de Botucatu Departamento de Medicina Interna Botucatu SP Brasil Departamento de Medicina Interna, Faculdade de Medicina de Botucatu, Universidade Estadual Paulista (UNESP), Botucatu, SP, Brasil; 2 Universidade Estadual Paulista Universidade Estadual Paulista Botucatu SP Brasil Universidade Estadual Paulista (UNESP), Botucatu, SP, Brasil; 3 Universidade de São Paulo Universidade de São Paulo Instituto de Ciências Biomédicas Departamento de Fisiologia e Biofísica São Paulo SP Brasil Departamento de Fisiologia e Biofísica, Instituto de Ciências Biomédicas, Universidade de São Paulo (USP), São Paulo, SP, Brasil

**Keywords:** Thyroid hormone, nongenomic actions, gene expression, breast cancer

## Abstract

**Objective:**

To verify the physiological action of triiodothyronine T_3_ on the expression of transforming growth factor α (*TGFA*) mRNA in MCF7 cells by inhibition of RNA Polymerase II and the MAPK/ERK pathway

**Materials and methods:**

The cell line was treated with T_3_ at a physiological dose (10^−9^M) for 10 minutes, 1 and 4 hour (h) in the presence or absence of the inhibitors, α-amanitin (RNA polymerase II inhibitor) and PD98059 (MAPK/ERK pathway inhibitor). *TGFA* mRNA expression was analyzed by RT-PCR. For data analysis, we used ANOVA, complemented with the Tukey test and Student t-test, with a minimum significance of 5%.

**Results:**

T_3_ increases the expression of *TGFA* mRNA in MCF7 cells in 4 h of treatment. Inhibition of RNA polymerase II modulates the effect of T_3_ treatment on the expression of *TGFA* in MCF7 cells. Activation of the MAPK/ERK pathway is not required for T_3_ to affect the expression of *TGFA* mRNA.

**Conclusion:**

Treatment with a physiological concentration of T_3_ after RNA polymerase II inhibition altered the expression of *TGFA*. Inhibition of the MAPK/ERK pathway after T_3_ treatment does not interfere with the *TGFA* gene expression in a breast adenocarcinoma cell line.

## INTRODUCTION

In the last few years, there has been a disturbing increase in the incidence of breast cancer (1), the second most common type of cancer and the fifth leading cause of cancer death worldwide (2). The thyroid hormone (TH) signaling pathway is complex and highly regulated by the expression of specific transporters of TH and multiple isoforms of the receptors of this hormone (TRs) present in several cells and by the interactions that occur between them (3,4). Besides its classic action via TR/elements responsive to TH (TERs), TH can act by other mechanisms (5); such mechanisms can be called non-classical or nongenomic action.

There is evidence that TH may affect the organization of the cytoskeleton, a mechanism by which it could interfere with the trafficking of vesicles to the membrane; this mechanism appears to involve αvβ3 integrin or a truncated TRα TH receptor that does not have a DNA binding domain and remains in the cytoplasm, where it can associate with other proteins/enzymes (3). From the interaction of TH with this integrin, the mitogen-activated protein kinase/extracellular signal-regulated kinase (MAPK/ERK) pathway is activated, which mediates the nongenomic effects of triiodothyronine (T_3_) on angiogenesis (6).

TH may act through non-classical or non-genomic mechanisms by interacting with iodothyronine receptors found on the plasma membrane or in the cytoplasm. The initiation sites are proteins characterized as iodothyronine receptors (7). The so-called non-genomic mechanisms may potentially influence gene expression, the onset of the pathway is non-genomic, as they depend on alternative pathways, but the consequences include increased transcription (5).

It is known that T_3_ plays a role in the development and progression of breast cancer, by inducing the expression of progesterone receptors and increasing the mRNA levels of proto-oncogenes, such as the transforming growth factor α (*TGFA*) (8,9).

*TGFA* can be produced by a variety of cells, primarily by cells of ectodermal origin and may interact with the Epidermal Growth Factor (EGF) receptor to induce growth and proliferation responses during cell contact (10). It is expressed in several tissues, from embryogenesis to the adult phase (11). *TGFA* has been described as a factor that is produced by tumor cells and is circumstantially implicated in the regulation of the autocrine growth of breast cancer cells (12,13); *TGFA* overexpression may occur during malignant progression (14). It has been reported that *TGFA* mRNA and protein can be detected in approximately 50-70% of breast tumors. In addition, 2-3 times higher levels of this biologically active factor can be found in the pleural effusions of patients with breast cancer (13).

Our group has shown that there is an increase in the expression of *TGFA* during T_3_ treatment (15), but this expression does not occur in cellular models that do not express the estrogen receptor or when the cells are simultaneously treated with antiestrogen medications, like tamoxifen (9,16).

For this study, we selected the MCF7 lineage of breast cancer cells. Since the 1980s, this cell line has been used as a model for breast tumors expressing estrogen receptor α (ERα), which is the main estrogen receptor (ER) expressed in this cell line and estrogen receptor β (ERβ) and also thyroid hormone receptor (TR) α and β (9,17–19).

The objective of the present study was to verify if the hormone T_3_, at a physiological concentration, activates the MAPK/ERK pathway (its inhibitor is PD98059) and RNA Polymerase II (its inhibitor is α-amanitin) to modulate the expression of *TGFA* gene in the MCF7 breast adenocarcinoma cell line in a short time period of 10 minutes (min), 1 and 4 hour (h) of treatment.

## MATERIALS AND METHODS

### Reagents

Roswell Park Memorial Institute (RPMI) 1640 medium, fetal bovine serum (FBS) and antibiotic solution at a 1:100 dilution were purchased from Gibco BRL (Grand Island, NY, USA). PD98059 (PD), α-amanitin, triiodothyronine (T_3_), dimethylsulfoxide (DMSO), sodium hydroxide (NaOH) and charcoal-stripped FBS were purchased from Sigma Aldrich (St Louis, MO, EUA).

### Cell culture

This project was approved by the Research Ethics Committee of Botucatu Medical School, protocol no. 3367-2009.

The MCF7 cell line, an eternal breast cancer cell line, was initially obtained from a primary culture of this cancer, developed from a pleural effusion of a female patient showing metastasis of the disease (20). These cells express both ERα and β as well as TRα and β (9,18,19). The cell line, initially acquired from the American Type Culture Collection (ATCC), Manassas, Virginia, USA, was expanded, grown and maintained in the cell bank of the Clinical Medicine Experimental Laboratory, UNESP, Botucatu. The cells were grown in RPMI 1640 medium supplemented with 1.2 g/L NaHCO_3_, 10 nM Hepes with pH 7.4 and 10% FBS and maintained at 37 °C in 5% CO_2_. The medium was changed every two days. To deplete all hormone sources in the culture medium, the cells were incubated with phenol red-free medium, supplemented with 10% charcoal-stripped FBS. After incubation, cells were treated with a physiological concentration T_3_, 10^-9^M, for 10 min, 1 h or 4 h, with all treatments being initiated at the same time, following which the cells were collected. The inhibitor and T_3_ hormone concentrations used were as follows: PD98059 (5 μM; T_3_ group associated with PD) was used as an inhibitor of the MAPK/ERK pathway and α-amanitin (50 μg/mL; T_3_ group associated with α-amanitin) was used as a transcription inhibitor. The untreated group received only 0.1% NaOH (T_3_ diluent) and served as the control (C). The inhibitors PD98059 and α-amanitin were added to the medium 1 h prior to the T_3_ treatment. The experiments were performed in triplicate.

### Gene expression

Total RNA was extracted from MCF7 cells using the Trizol method (Invitrogen, São Paulo, Brazil), according to the manufacturer's instructions. The High-Capacity cDNA Reverse Transcription RT-PCR kit (Invitrogen, São Paulo, Brazil) was used for the synthesis of 20 μL of complementary DNA (cDNA) from 1000 ng of total RNA. *TGFA* levels (Hs00608187_m1, Applied Biosystems, Foster City, CA) were determined by Real-Time Quantitative Reverse Transcription PCR (qRT-PCR). The quantitative measurements were performed with the Applied Biosystems StepOne Plus detection system using the commercial TaqMan kit for qPCR (Applied Biosystems), according to the manufacturer's instructions. The amplification conditions were activation of the enzyme at 50°C for 2 min, denaturation at 95°C for 10 min and amplification for 40 cycles of denaturation at 95°C for 15 s and extension at 60°C for 1 min. The analyses were performed in duplicate. Gene expression was quantified relative to the values of the control group, after normalization of the expression to that of an internal control, GAPDH (Hs02758991_g1), by the method of 2^–ΔΔCt^, as previously described (21).

### Statistical analysis

For statistical analysis, ANOVA was used in conjunction with the Tukey test and Student's t-test and a minimum significance of 5% was assumed. Data were expressed as the mean ± standard deviation.

## RESULTS

### T_3_ increases the expression of *TGFA mRNA* in MCF7 cells in 4 h

**Figure 1:**
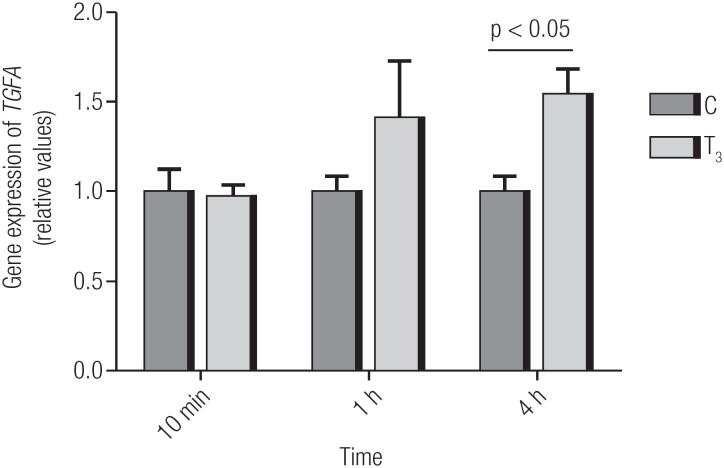
Effects of T_3_ treatment length on *TGFA* mRNA levels in MCF7 cells. C: Control; T_3_: Triiodothyronine. The treatment times were 10 min, 1 h and 4 h. Relative expression levels represent the mean of 3 replicates. Statistical analysis was performed using a Student's t-test; p<0.05 is considered significant.

There was a significant increase in the gene expression of *TGFA* after the treatment of MCF7 cells with T_3_ for 4 h, but the increase was not observed after the treatment time-points of 10 min and 1 h (Figure 1). Comparison CxT_3_.

### Effect of T_3_ on the maintenance of *TGFA* gene expression after inhibition of RNA polymerase II in MCF7 cells

**Figure 2:**
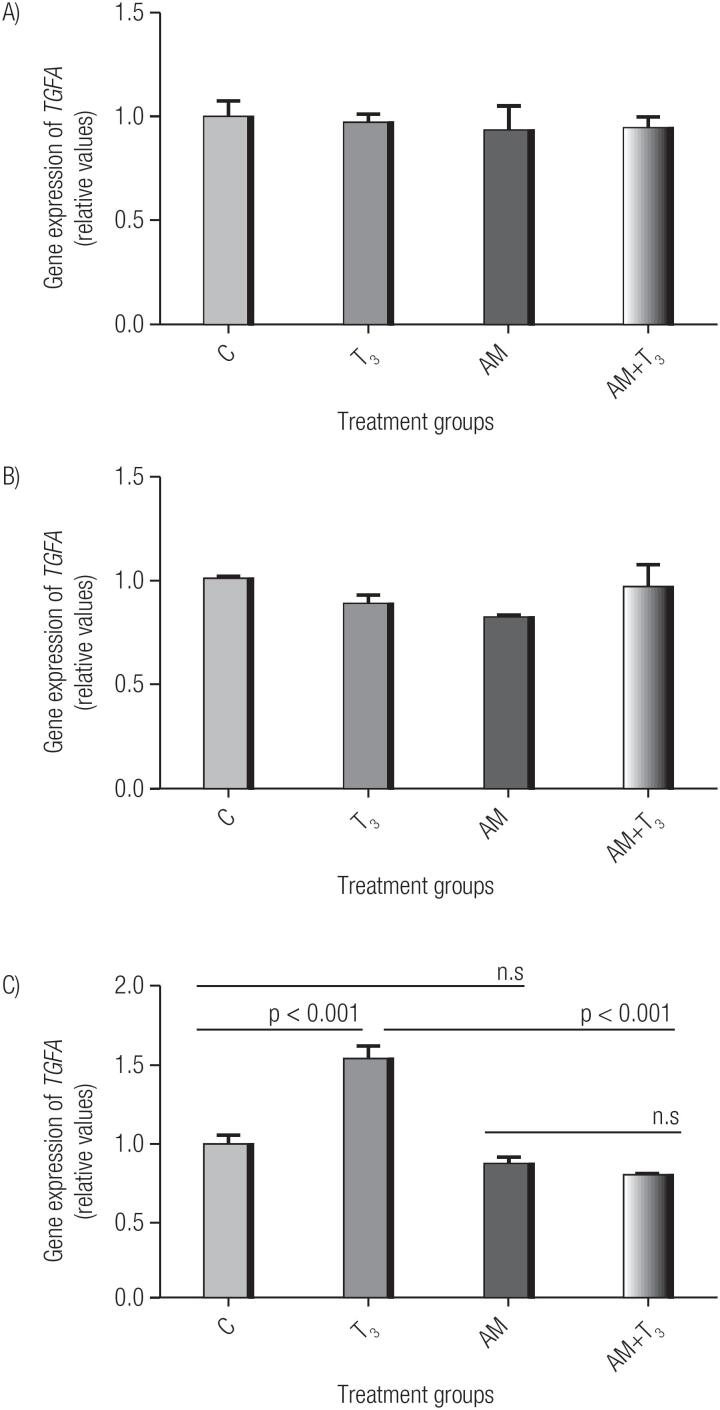
Effect of T_3_ in association with α-amanitin (AM) on the gene expression of *TGFA* in MCF7 cells. C: Control; T_3_: Triiodothyronine; AM: α-amanitin; AM+T_3_: α-amanitin associated with triiodothyronine. Triiodothyronine treatment was performed for 10 min (A), 1 h (B) and 4 h (C). Relative expression levels represent the mean of 3 replicates. Statistical analysis was performed using an ANOVA, supplemented with the Tukey test; p<0.05 is considered significant.

After 4 h of treatment, the T_3_ group associated with α-amanitin showed a significant decrease in *TGFA* gene expression, compared to the T_3_ group. However, there was no change in the expression when comparing the *TGFA* expression in the α-amanitin group with that in the control group and in the T_3_ group associated with α-amanitin (Figure 2). Comparison CxT_3_, CxAM, T_3_xAM+T_3_ and AMxAM+T_3_.

### Activation of the MAPK/ERK pathway is not required for T_3_ to affect the expression of *TGFA mRNA*

**Figure 3:**
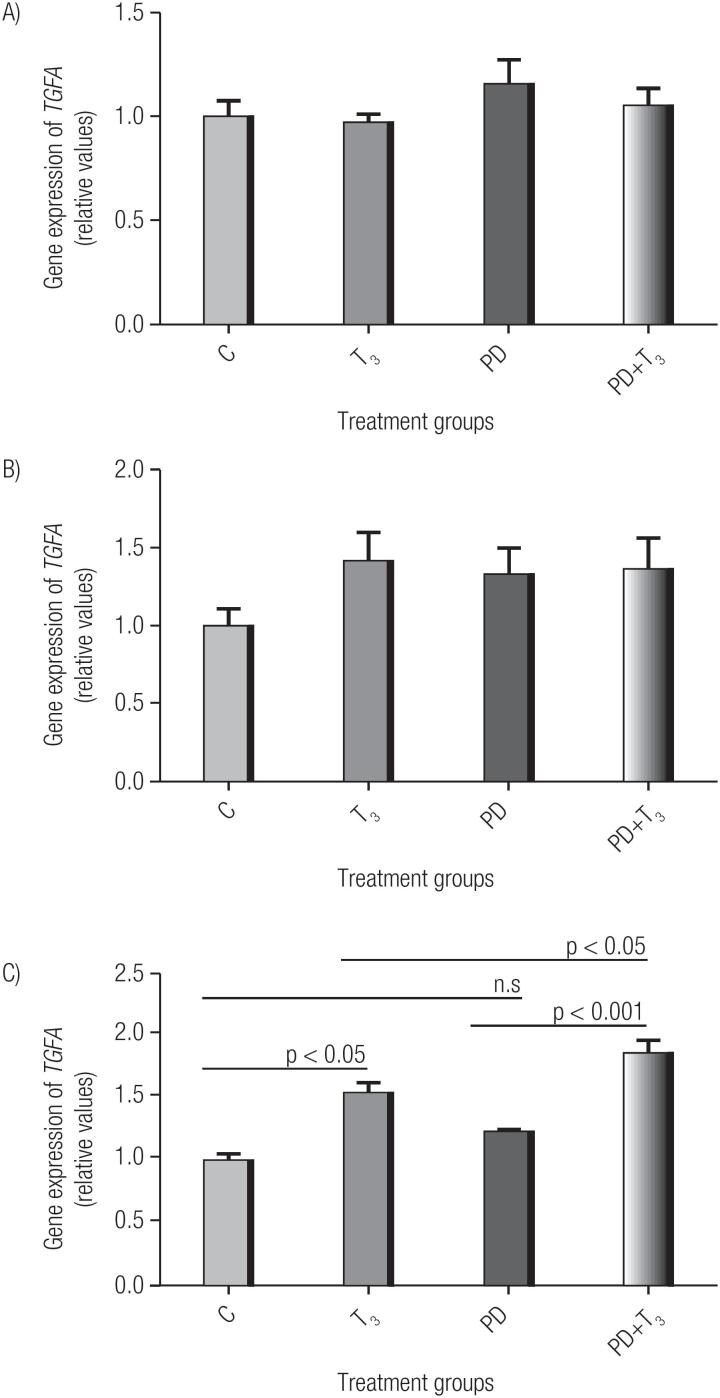
Effect of T_3_ in association with PD98059 (PD) on the gene expression of *TGFA* in MCF7 cells. C: Control; T_3_: Triiodothyronine; PD: PD98059; PD+T_3_: PD98059 associated with triiodothyronine. Triiodothyronine treatment was performed for 10 min (A), 1 h (B) and 4 h (C). Relative expression levels represent the mean of 3 replicates. Statistical analysis was performed using an ANOVA, supplemented with the Tukey test; p<0.05 is considered significant.

After 4 h of treatment, the T_3_ group associated with PD showed a significant increase in *TGFA* gene expression, compared to the T_3_ group and the PD group. However, there was no change in this expression when we compared the *TGFA* expression in the PD and control groups (Figure 3). Comparison CxT_3_, CxPD, T_3_xPD+T_3_ and PDxPD+T_3_.

## DISCUSSION

In the breast cancer cell line MCF7, T_3_ regulates gene expression either by binding to specific TRs that bind to TREs in the promoter region of target genes (22–25), or by activating alternative pathways, such as the MAPK/ERK pathway, via their initiation sites, which are present in the plasma membrane or in the cytoplasm (7).

Our group identified an increase in the expression of *TGFA* (indicative of increased proliferative activity) in primary breast cancer cultures following treatment with estrogen and T_3_ (16). Moretto and cols. (15) demonstrated an increased *TGFA* expression in MCF7 cells after treatment with supraphysiological concentrations of T_3_ (10^-8^M) over periods of 10 min, 30 min, 1 h, and 4 h. In the present study, we show that treatment with physiological concentrations of T_3_ (10^-9^M) also increases the expression of *TGFA*, corroborating the results obtained by Moretto and cols.; however, at this lower dosage we were unable to detect expression changes in treatments under 4 h.

Fernandez and cols. (26) studied the effects of triiodothyronine on MDA-468 breast cancer cells, showing that at physiological doses, the hormone exerts control over the actions of *TGFA.* Unlike these studies, we show that short treatments with physiological concentrations of T_3_ do not affect *TGFA* expression. Brito and cols. (27) demonstrated that *TGFA* expression is increased in esophageal cancer, indicating differential regulation of the gene in esophageal tumorigenesis.

α-amanitin is a general transcription inhibitor that is selective for RNA polymerase II and is approved for the treatment of some types of sarcomas (28,29). We have shown that on exposure to physiological T_3_ concentrations, there was an increase in the *TGFA* expression in a breast cancer cell line after 4 h, which suggests that further studies should be done to determine the effects of transcription blocking drugs on breast adenocarcinoma cell lines. Our data suggest that α-amanitin may provide additional therapeutic benefits when used in combination with other anticancer agents. This could allow for the development of more specific and effective drugs in the treatment of breast adenocarcinomas.

We determined that T_3_-mediated inhibition of the MAPK/ERK pathway leads to overexpression of *TGFA*. Studies have reported that increased expression of EGFR family proteins are a poor prognostic factor for cancer patients (30–32); is also associated with tumor aggressiveness (32) and resistance to chemotherapy (30) and their levels are increased in 30% of solid tumors (31). Hence, we can infer that as the MAPK/ERK pathway is not modulated by T_3_ treatment, this hormone can use some precursor that induces increased tumorigenesis in MCF7 cells. This situation was previously described in a report by Boldt and cols. (33), who demonstrated that the modulation of the MAPK/ERK pathway, as an adjuvant treatment for breast cancer, is of limited value, as it may increase or decrease the efficacy of other chemotherapeutic drugs.

We conclude that the treatment of MCF7 cells with physiological doses of the hormone T_3_, over a period of 4 h, leads to an increased expression of *TGFA* via RNA Polymerase II. We also conclude that T_3_ does not increase *TGFA* expression by acting on the MAPK/ERK pathway.

The results obtained in this study, together with previous results from our research group, suggest that T_3_ acts via nuclear pathways when present at physiological doses and via extra-nuclear pathways when present at supraphysiological doses.

Further studies are required to explore the effects of the modulation of the *TGFA* proto-oncogene in the treatment of breast adenocarcinomas.
